# Solitary wave solutions of the time fractional Benjamin Bona Mahony Burger equation

**DOI:** 10.1038/s41598-024-65471-w

**Published:** 2024-06-25

**Authors:** K. Pavani, K. Raghavendar, K. Aruna

**Affiliations:** Department of Mathematics, School of Advanced Sciences, Vellore Institute of Technology, Vellore, 632014 India

**Keywords:** Benjamin Bona Mahony Burger equation, Atangana Baleanu Caputo derivative, Caputo Fabrizio derivative, Caputo derivative, Mathematics and computing, Applied mathematics

## Abstract

The present study examines the approximate solutions of the time fractional Benjamin Bona Mahony Burger equation. This equation is critical for characterizing the dynamics of water waves and fluid acoustic gravity waves, as well as explaining the unidirectional propagation of long waves in nonlinear dispersive systems. This equation also describes cold plasma for hydromagnetic and audio waves in harmonic crystals. The natural transform decomposition method is used to obtain the analytical solution to the time fractional Benjamin Bona Mahony Burger equation. The proposed method uses the Caputo, Caputo Fabrizio, and Atangana Baleanu Caputo derivatives to describe the fractional derivative. We utilize a numerical example with appropriate initial conditions to assess the correctness of our findings. The results of the proposed method are compared to those of the exact solution and various existing techniques, such as the fractional homotopy analysis transform method and the homotopy perturbation transform technique. As a result, bell shaped solitons are discovered under the influence of hyperbolic functions. By comparing the outcomes with tables and graphs, the findings demonstrate the efficacy and effectiveness of the suggested approach.

## Introduction

Fractional calculus (FC) is an evolving topic that is gaining popularity in several areas of engineering research. Fractional differential equations (FDEs) have become significant and well-liked, mostly because of their proven utility in the fields of science and engineering. Researchers are increasingly utilizing these equations to model problems in various research fields, including dynamical systems, mechanical systems, control, chaos, chaos synchronization, anomalous diffusive and sub-diffusive systems, wave propagation phenomena, epidemiology, and infectious diseases^1–6^. The adaptability of fractional theory and its many fascinating aspects have drawn the attention of a number of academics. FC is used to handle complex natural phenomena with the definitions of fractional derivatives such as Caputo (C), Caputo-Hadamard, Riemann–Liouville (R–L), Caputo Fabrizio (CF), Erdélyi–Kober, and Atangana Baleanu Caputo (ABC). Among the most commonly used fractional derivatives in several studies over the last few years have been the C, CF, and ABC. The Caputo derivative has a singular kernel. A novel fractional differential operator that Caputo and Fabrizio introduced has the benefit of a non-singular kernel. This new operator has piqued the interest of many researchers and has found application in numerous model equations. This operator is non-local, however, and has a drawback in that the related integral is not a fractional operator. To overcome this drawback, Atangana and Baleanu developed a new fractional operator in Caputo and R–L based on the Mittag–Leffler function. Researchers have studied numerous physical issues using this operator, which has also attracted interest. The formulation of a number of electrochemical issues has been shown to benefit more from integrals and non-integer-order derivatives than from conventional approaches^7^.

The primary benefit of employing FDEs in modeling realistic applications lies in their non-local properties. The local nature of the integer-order differential operator is widely recognized, but the fractional order differential operator is characterized by its non-local behavior. This indicates that the future state of a system is influenced not just by its present state, but also by all of its past states. Due to its increased realism, fractional calculus has gained popularity. Fractional partial differential equations (FPDEs) are a potent modeling tool widely used in physics, engineering, and applied sciences. They have a broad range of applications and may describe various evolving events. The calculation that employs operators for differentiation and integration with non-integer orders is referred to as the conventional extension of the computation. In both technological and theoretical disciplines, they are regarded as useful and effective instruments for addressing the nonlinearity and complexity of certain issues, such as those in fluid flow, signaling systems, chemical research, device recognition, fiber optics, polymers, and elastic materials^8,9^. In recent years, a number of researchers working on these issues have focused their efforts on investigating the solutions of nonlinear PDEs using a variety of methodologies such as q-homotopy analysis transform method^10–12^, q-homotopy analysis Elzaki transform method^13^, Laplace variational iteration scheme^14^, homotopy perturbation with the Sumudu transformation approach^15^, modified fractional differential transform approach^16^, residual power series approach^17^, Shehu transform decomposition method^18,19^, and operational matrix technique^20^.

Consider the nonlinear time fractional Benjamin Bona Mahony Burger equation (TFBBMBE)^21^ as follows:

1 $$\begin{aligned} D_{\tau }^{\mu }u-u_{\zeta \zeta \tau }+uu_{\zeta }+u_{\zeta }=0, ~ 0<\mu \le 1,~ \tau >0, \end{aligned}$$

with the initial condition

2 $$\begin{aligned} u(\zeta ,0)=\textrm{sech}^{2}\Big (\frac{\zeta }{4}\Big ). \end{aligned}$$

The exact solution of TFBBMBE, when $\mu =1$ is given by

3 $$\begin{aligned} u(\zeta ,\tau )=\textrm{sech}^2\Big (\frac{\zeta }{4}-\frac{\tau }{3}\Big ), \end{aligned}$$

where $u(\zeta ,\tau )$ represents the fluid velocity in the horizontal direction, and $\mu$ represents the fractional order derivative. The Benjamin Bona Mahony Burger equation^22^, which describes a variety of unique wave events, has important applications in the field of ocean engineering. Additionally, the study may make it possible for other experts in the field to investigate the role that dispersive waves play in coastal processes in more detail. The ocean wave model is based on the Benjamin Bona Mahony Burger equation. Despite numerous challenges in the mathematical analysis, the Benjamin Bona Mahony Burger equation has gained widespread recognition for its capacity to implement mathematical models for the long-term evolution of nonlinear wave events. TFBBMBE, which represents the unidirectional propagation of tiny, long waves on the water surface in a channel, is also an alternative to the Korteweg-de Vries (KdV) equation. Compared to the KdV equation, the TFBBMBE offers greater benefits, as it can predict not only shallow water waves but also hydromagnetic waves in cold plasma. TFBBMBEs have a wide range of applications, including shattered rock, sound waves in harmonic crystals^23^, thermodynamics, acoustic-gravity waves in liquids, etc. Due to its vast applications in science and engineering, several researchers have used various methods to examine the exact and approximate solutions of the TFBBMBE. We list a few methods, namely homotopy perturbation transform technique (HPTT)^21^, modified residual power series method^24^, Crank–Nicolson linear difference technique^25^, linearized difference technique^26^, homotopy analysis method^27^, fractional homotopy analysis transform method (FHATM)^28^, and $\Big (\frac{G^{\prime }}{G}\Big )$ expansion method^29^, and finite element method^30^.

To the best of the authors knowledge, this is the first attempt to use natural transform decomposition method (NTDM) to find the approximate solutions to TFBBMBE by taking the fractional derivative in singular and non-singular kernel derivatives. Rawashdeh and Maitama^31^ proposed NTDM to find approximate solutions for a class of nonlinear PDEs. For the relationship between the natural transform and other integral transforms, we refer to^32^ and references therein. The Adomian decomposition and natural transform (NT) are combined to develop the NTDM, which is a highly effective approach. Some kind of coupled nonlinear PDEs can be easily and quickly solved with this novel approach, which is considered a powerful tool. Given that it employs a fast convergence series, the answer that this method provides may be exact or approximate. Round-off errors can be effectively reduced by the NTDM without the need for linearization, discretization, prescribed assumptions, or perturbation. The homotopy perturbation method has a basic drawback in that each iteration requires solving the functional equation, which can be challenging and time-consuming. The variational iteration method necessitates the use of Lagrange’s multiplier to calculate the correction functional. However, it faces challenges in satisfying the stationary conditions in every situation. The suggested method does away with the necessity to compute fractional derivatives or fractional integrals in the recursive formula, unlike the traditional Adomian method. The capacity to compute the terms values in the series is improved by this modification. Identification of the parameter *h* is a difficult step in the homotopy analysis approach. However, the NTDM has a straightforward approach and solves nonlinear fractional differential equations with great efficiency.

This framework is efficacious for ascertaining the analytical solution to fractional order partial differential equations, but only under specific initial conditions in time. According to our comprehension, the proposed method seems feasible for solving initial value problems. However, it may not be suitable for problems that involve boundary constraints. Thus, the proposed method is specifically designed for transient state problems that offer the initial distribution of quantities. The NTDM is used to investigate several physical phenomena of FPDEs, including the analytical model of a few non-linear partial differential equation systems and the presentation of additional physical phenomena. The proposed technique was recently used to solve time fractional differential equations such as the Kawahara and modified Kawahara equations^33^, Zakharov–Kuznetsov equation^34^, Swift-Hohenberg equation^35^, Klein–Gordon equation^36^, and coupled system of shallow water equations^37^. To sum up, the proposed method is advantageous for academics and practitioners alike in terms of accuracy, adaptability, and numerical efficiency. It also helps in capturing non-local influences and improves comparative analysis abilities and practicality.

The following is the structure of the manuscript: “Preliminaries” covers the fundamental concepts of fractional derivatives and their natural transforms. The basic idea of NTDM to be employed on the proposed model utilizing singular and nonsingular kernel derivatives is presented in “Description of the NTDM”. Section “Convergence analysis” discusses convergence analysis and uniqueness. The outcomes of the proposed study are illustrated with a numerical example of TFBBMBE in “Numerical example of TFBBMBE”. Results and discussions are provided in “Results and discussion”. Section “Conclusion” summarizes the conclusions of the presented work .

## Preliminaries

In this section, we present definitions of fractional derivatives along with their natural transform.

###  Definition 2.1^7^

The Caputo derivative of $f\in C_{-1}^{q}$ is given by

4 $$\begin{aligned} D^{\mu }_{\tau }f(\tau )= {\left\{ \begin{array}{ll} \frac{d^{q}f(\tau )}{d\tau ^{q}}, ~~ \mu =q\in \mathbb {N},\\ \frac{1}{\Gamma (q-\mu )}\int _{0}^{\tau }(\tau -\xi )^{q-\mu -1}f^{q}(\xi )d\xi , ~q-1<\mu \leqslant q,~ q\in \mathbb {N}. \end{array}\right. } \end{aligned}$$

###  Definition 2.1^38^

The CF derivative of *f* with order $\mu$ is defined as

5 $$\begin{aligned} {}^{CF}_{}D_{\tau }^{\mu }f(\tau )=\frac{1}{1-\mu }\int _{0}^{\tau }f^{\prime }(\xi )~ \textrm{exp}\Bigg (\frac{-\mu (\tau -\xi )}{1-\mu }\Bigg ) d\xi ,~\tau \ge 0. \end{aligned}$$

###  Definition 2.2^39^

The ABC fractional derivative of *f* is given by

6 $$\begin{aligned} {}^{ABC}_{}D_{\tau }^{\mu }f(\tau )=\frac{M[\mu ]}{1-\mu }\int _{0}^{\tau }f^{\prime }(\xi ) E_{\mu }\Bigg (\frac{-\mu (\tau -\xi )^{\mu }}{1-\mu }\Bigg ) d\xi , 0<\mu <1, \end{aligned}$$

where the Mittag–Leffler function is $E_{\mu }$.

### Definition 2.3^40^

The NT definition of $f(\vartheta )$ is given as

7 $$\begin{aligned} N^{+}[f(\vartheta )]=R(s,w)=\int _{0}^{\infty } e^{-s\tau }f(\vartheta )d\vartheta , ~~w,s> 0. \end{aligned}$$

### Definition 2.4^41^

The NT of ${}^{C}_{0}D_{\tau }^{\mu }w(\vartheta )$ in terms of C is given as

8 $$\begin{aligned} N^{+}[{}^{C}_{0}D_{\tau }^{\mu }w(\vartheta )]= \Bigg (\frac{s}{v}\Bigg )^{\mu }\Bigg (N^{+}[w(\vartheta )]-\frac{1}{s}w(0)\Bigg ). \end{aligned}$$

### Definition 2.5^42^

The NT of ${}^{CF}_{0}D_{\tau }^{\mu }w(\vartheta )$ in terms of CF is given as

9 $$\begin{aligned} N^{+}[{}^{CF}_{0}D_{\tau }^{\mu }w(\vartheta )]= \frac{1}{g(\mu ,s,v)}\Bigg (N^{+}[w(\vartheta )]-\frac{1}{s}w(0)\Bigg ), \end{aligned}$$

where $$g(\mu ,s,v)=1-\mu +\mu (\frac{v}{s})$$.

### Definition 2.6^43^

The NT of ${}^{ABC}_{0}D_{\tau }^{\mu }w(\vartheta )$ in terms of ABC is given as

10 $$\begin{aligned} N^{+}[{}^{ABC}_{0}D_{\tau }^{\mu }w(\vartheta )]= \frac{1}{h(\mu ,s,v)}\Bigg (N^{+}[w(\vartheta )]-\frac{1}{s}w(0)\Bigg ), \end{aligned}$$

where $h(\mu ,s,v)=\frac{1-\mu +\mu (\frac{v}{s})^{\mu }}{M[\mu ]}$, and the normalization function is $M[\mu ]$.

## Description of the NTDM

This section describes the procedure of NTDM to solve TFBBMBE.

**NTDM**$_{C}$: By applying NT to (1) in terms of the C fractional derivative, we obtain

11 $$\begin{aligned} \Big (\frac{s}{v}\Big )^{\mu }\left[ N^{+}(u(\zeta ,\tau ))-\frac{\textrm{sech}^{2}\Big (\frac{\zeta }{4}\Big )}{s}\right] =N^{+}[u_{\zeta \zeta \tau }-uu_{\zeta }-u_{\zeta }]. \end{aligned}$$

On employing inverse NT on (11), we have

12 $$\begin{aligned} u(\zeta ,\tau )=N^{-1}\left[ \frac{\textrm{sech}^{2}\Bigg (\frac{\zeta }{4}\Bigg )}{s}+\Bigg (\frac{v}{s}\Bigg )^{\mu }N^{+}\Bigg [u_{\zeta \zeta \tau }-uu_{\zeta }-u_{\zeta }\Bigg ]\right] . \end{aligned}$$

The nonlinear term can be expressed as

13 $$\begin{aligned} uu_{\zeta }=\sum _{k=0}^{\infty }A_{k}, \end{aligned}$$

where $A_{k}$ represents the Adomian polynomials. Assume that $u(\zeta , \tau )$ can have the following infinite series form:

14 $$\begin{aligned} u(\zeta , \tau ) =\sum _{k=0}^{\infty } u_{k}(\zeta , \tau ). \end{aligned}$$

Now, making use of the Eqs. (14) and (13) into (12), we obtain

15 $$\begin{aligned} \begin{aligned} \sum _{k=0}^{\infty } u_{k}(\zeta ,\tau )&=N^{-1}\left[ \frac{\textrm{sech}^{2}\Big (\frac{\zeta }{4}\Big )}{s}\right] \\&\quad +N^{-1}\left[ \Big (\frac{v}{s}\Big )^{\mu }N^{+}\left[ \sum _{k=0}^{\infty }(u_k)_{\zeta \zeta \tau }-\sum _{k=0}^{\infty }\sum _{j=0}^{k}u_{j}(u_{k-j})_{\zeta }-\sum _{k=0}^{\infty }(u_{k})_{\zeta }\right] \right] . \end{aligned} \end{aligned}$$

From (15), we have

$$\begin{aligned} {}^{C}u_{0}(\zeta ,\tau )&=N^{-1}\left[ \frac{\textrm{sech}^{2}\Big (\frac{\zeta }{4}\Big )}{s}\right] ,\\ {}^{C}u_{1}(\zeta ,\tau )&= N^{-1}\Big [\Big (\frac{v}{s}\Big )^{\mu }N^{+}\Big [u_{0\zeta \zeta \tau }-u_{0}u_{0\zeta }-u_{0\zeta }\Big ]\Big ],\\ {}^{C}u_{2}(\zeta ,\tau )&= N^{-1}\Big [\Big (\frac{v}{s}\Big )^{\mu }N^{+}\Big [u_{1\zeta \zeta \tau }-(u_{0}u_{1\zeta }+u_{1}u_{0\zeta })-u_{1\zeta }\Big ]\Big ],\\&\vdots \end{aligned}$$

16 $$\begin{aligned} {}^{C}u_{k+1}(\zeta ,\tau )= N^{-1}\left[ \Big (\frac{v}{s}\Big )^{\mu }N^{+}\left[ (u_{k})_{\zeta \zeta \tau }-\sum _{j=0}^{k}u_{j}(u_{k-j})_{\zeta }-(u_{k})_{\zeta }\right] \right] ,~ k\ge 0. \end{aligned}$$

Finally, the NTDM$_{C}$ solution is obtained by substituting the Eq. (16) into (14) as shown below:

17 $$\begin{aligned} {}^{C}u(\zeta ,\tau )={}^{C}u_{0}(\zeta ,\tau )+{}^{C}u_{1}(\zeta ,\tau )+{}^{C}u_{2}(\zeta ,\tau )+\dots ~. \end{aligned}$$

**NTDM**$_{CF}$: By applying NT to (1) in terms of the CF fractional derivative, we obtain

18 $$\begin{aligned} \frac{1}{g(\mu ,s,v)}\left[ N^{+}(u(\zeta ,\tau ))-\frac{\textrm{sech}^{2}\Big (\frac{\zeta }{4}\Big )}{s}\right] =N^{+}[u_{\zeta \zeta \tau }-uu_{\zeta }-u_{\zeta }]. \end{aligned}$$

On employing inverse NT on (18), we get

19 $$\begin{aligned} u(\zeta ,\tau )=N^{-1}\left[ \frac{\textrm{sech}^{2}\Big (\frac{\zeta }{4}\Big )}{s}+g(\mu ,s,v)N^{+}\left[ u_{\zeta \zeta \tau }-uu_{\zeta }-u_{\zeta }\right] \right] . \end{aligned}$$

Now, making use of the Eqs. (14) and (13) into (19), we obtain

20 $$\begin{aligned} \begin{aligned} \sum _{k=0}^{\infty } u_{k}(\zeta ,\tau )&=N^{-1}\left[ \frac{\textrm{sech}^{2}\Big (\frac{\zeta }{4}\Big )}{s}\right] \\&\quad +N^{-1}\left[ g(\mu ,s,v)N^{+}\left[ \sum _{k=0}^{\infty }(u_k)_{\zeta \zeta \tau }-\sum _{k=0}^{\infty }\sum _{j=0}^{k}u_{j}(u_{k-j})_{\zeta }-\sum _{k=0}^{\infty }(u_{k})_{\zeta }\right] \right] . \end{aligned} \end{aligned}$$

From (20), we have

$$\begin{aligned} {}^{CF}u_{0}(\zeta ,\tau )&=N^{-1}\left[ \frac{\textrm{sech}^{2}\Big (\frac{\zeta }{4}\Big )}{s}\right] ,\\ {}^{CF}u_{1}(\zeta ,\tau )&= N^{-1}\Big [g(\mu ,s,v)N^{+}\Big [u_{0\zeta \zeta \tau }-u_{0}u_{0\zeta }-u_{0\zeta }\Big ]\Big ],\\ {}^{CF}u_{2}(\zeta ,\tau )&= N^{-1}\Big [g(\mu ,s,v)N^{+}\Big [u_{1\zeta \zeta \tau }-(u_{0}u_{1\zeta }+u_{1}u_{0\zeta })-u_{1\zeta }\Big ]\Big ],\\&\vdots \end{aligned}$$

21 $$\begin{aligned} {}^{CF}u_{k+1}(\zeta ,\tau )= N^{-1}\left[ g(\mu ,s,v)N^{+}\left[ (u_{k})_{\zeta \zeta \tau }-\sum _{j=0}^{k}u_{j}(u_{k-j})_{\zeta }-(u_{k})_{\zeta }\right] \right] ,~ k\ge 0. \end{aligned}$$

Finally, the NTDM$_{CF}$ solution is obtained by substituting the Eq. (21) into (14) as shown below:

22 $$\begin{aligned} {}^{CF}u(\zeta ,\tau )={}^{CF}u_{0}(\zeta ,\tau )+{}^{CF}u_{1}(\zeta ,\tau )+{}^{CF}u_{2}(\zeta ,\tau )+\dots ~. \end{aligned}$$

**NTDM**$_{ABC}$: By applying NT to (1) in terms of the ABC fractional derivative, we obtain

23 $$\begin{aligned} \frac{1}{h(\mu ,s,v)}\left[ N^{+}(u(\zeta ,\tau ))-\frac{\textrm{sech}^{2}\Big (\frac{\zeta }{4}\Big )}{s}\right] =N^{+}[u_{\zeta \zeta \tau }-uu_{\zeta }-u_{\zeta }]. \end{aligned}$$

On employing inverse NT on (23), we get

24 $$\begin{aligned} u(\zeta ,\tau )=N^{-1}\left[ \frac{\textrm{sech}^{2}\Big (\frac{\zeta }{4}\Big )}{s}+h(\mu ,s,v)N^{+}\Big [u_{\zeta \zeta \tau }-uu_{\zeta }-u_{\zeta }\Big ]\right] . \end{aligned}$$

Now, making use of the Eqs. (14) and (13) into (24), we obtain

25 $$\begin{aligned} \begin{aligned} \sum _{k=0}^{\infty } u_{k}(\zeta ,\tau )&=N^{-1}\left[ \frac{\textrm{sech}^{2}\Big (\frac{\zeta }{4}\Big )}{s}\right] \\&\quad +N^{-1}\Big [h(\mu ,s,v)N^{+}\Big [\sum _{k=0}^{\infty }(u_k)_{\zeta \zeta \tau }-\sum _{k=0}^{\infty }\sum _{j=0}^{k}u_{j}(u_{k-j})_{\zeta }-\sum _{k=0}^{\infty }(u_{k})_{\zeta }\Big ]\Big ]. \end{aligned} \end{aligned}$$

From (25), we have

$$\begin{aligned} {}^{ABC}u_{0}(\zeta ,\tau )&=N^{-1}\left[ \frac{\textrm{sech}^{2}\Big (\frac{\zeta }{4}\Big )}{s}\right] ,\\ {}^{ABC}u_{1}(\zeta ,\tau )&= N^{-1}\left[ h(\mu ,s,v)N^{+}\left[ u_{0\zeta \zeta \tau }-u_{0}u_{0\zeta }-u_{0\zeta }\right] \right] ,\\ {}^{ABC}u_{2}(\zeta ,\tau )&= N^{-1}\Big [h(\mu ,s,v)N^{+}\Big [u_{1\zeta \zeta \tau }-(u_{0}u_{1\zeta }+u_{1}u_{0\zeta })-u_{1\zeta }\Big ]\Big ],\\&\vdots \end{aligned}$$

26 $$\begin{aligned} {}^{ABC}u_{k+1}(\zeta ,\tau )= N^{-1}\left[ h(\mu ,s,v)N^{+}\left[ (u_{k})_{\zeta \zeta \tau }-\sum _{j=0}^{k}u_{j}(u_{k-j})_{\zeta }-(u_{k})_{\zeta }\right] \right] ,~ k\ge 0. \end{aligned}$$

Finally, the NTDM$_{ABC}$ solution is obtained by substituting the Eq. (26) into (14) as shown below:

27 $$\begin{aligned} {}^{ABC}u(\zeta ,\tau )={}^{ABC}u_{0}(\zeta ,\tau )+{}^{ABC}u_{1}(\zeta ,\tau )+{}^{ABC}u_{2}(\zeta ,\tau )+\cdots ~. \end{aligned}$$

## Convergence analysis

In this section, we establish the necessary conditions that ensure the existence of a unique solution and address the convergence of the solution.

### Theorem 1

^36^ The proposed technique approximate solution with respect to Caputo derivative ($NTDM_{C}$) is unique when $0<(\theta _1+\theta _2)\frac{\tau ^{\mu }}{\Gamma (\mu +1)}<1$.

### Proof

Let $E=(C[I],\left\Vert .\right\Vert )$, here the norm $\left\Vert \phi (\tau )\right\Vert =\max \limits _{\tau \in I}|\phi (\tau )|$ is a Banach space, and continuous function on *I*. Let the nonlinear mapping $R:E\rightarrow E$, where

$$\begin{aligned} u_{k+1}^{C}(\zeta ,\tau )=u_0^{C}+N^{-1}\Big [\Big (\frac{v}{s}\Big )^{\mu }N^{+}[L(u_k(\zeta ,\tau ))+G(u_k(\zeta ,\tau ))]\Big ], k\ge 0. \end{aligned}$$

Here *L* and *G* are the linear and nonlinear terms. Let us suppose $\mid L(u)-L(u^*) \mid <\theta _1\mid u-u^*\mid$ and $\mid G(u)-G(u^*)\mid <\theta _2\mid u-u^*\mid ,$ where *u* and $u^{*}$ are two various values of the function and $\theta _1$, $\theta _2$ are two Lipschitz constants.

$$\begin{aligned} \begin{aligned} \left\Vert R(u)-R(u^*)\right\Vert&\le \max _{\tau \in I}\Big |N^{-1}\Big [\Big (\frac{v}{s}\Big )^{\mu }N^{+}[L(u)-L(u^*)] +g(\mu ,s,v)N^{+}[G(u)-G(u^*)]\Big ]\Big |\\&\le \max _{\tau \in I}\Big [\theta _1 N^{-1}\Big [\Big (\frac{v}{s}\Big )^{\mu }N^{+}| u-u^*|\Big ]+\theta _2 N^{-1}\Big [\Big (\frac{v}{s}\Big )^{\mu }N^{+}| u-u^*|\Big ]\Big ]\\&\le \max _{\tau \in I} (\theta _1+\theta _2)\Big [N^{-1}\Big [\Big (\frac{v}{s}\Big )^{\mu }(N^{+}| u-u^*|)\Big ]\Big ]\\&\le (\theta _1+\theta _2)\Big [N^{-1}\Big [\Big (\frac{v}{s}\Big )^{\mu }N^{+}\left\Vert u-u^*\right\Vert \Big ]\Big ]\\&=(\theta _1+\theta _2)\frac{\tau ^{\mu }}{\Gamma (\mu +1)}\left\Vert u-u^*\right\Vert . \end{aligned} \end{aligned}$$

Under the condition of $0<(\theta _1+\theta _2)\frac{\tau ^{\mu }}{\Gamma (\mu +1)}<1$, the mapping is contraction. The uniqueness of the solution to Eq. (1) can be established with the use of the Banach fixed point theorem. $\square$

By following the above Theorem 1 procedure in a similar way, we can prove that $NTDM_{CF}$ and $NTDM_{ABC}$ approximate solutions are unique.

### Theorem 2

^36^ $NTDM_{C}$ solution is convergent.

### Proof

Let $u_p=\sum \nolimits _{k=0}^{p}u_k(\zeta ,\tau )$. In Banach space *E*, we illustrate that $u_{p}$ is a Cauchy sequence.

Now,

$$\begin{aligned} \begin{aligned} \left\Vert u_p-u_q\right\Vert&=\max _{\tau \in I}\left| \sum _{r=q+1}^{p}u_r\right| ,~ q=1,2,3,~ \dots ~.\\&\le \max _{\tau \in I}\left| N^{-1}\left[ \Big (\frac{v}{s}\Big )^{\mu }N^{+}\left[ \sum _{r=q+1}^{p}(L(u_{r-1})+G(u_{r-1}))\right] \right] \right| \\&=\max _{\tau \in I}\left| N^{-1}\left[ \Big (\frac{v}{s}\Big )^{\mu }N^{+}\left[ \sum _{r=q}^{p-1}L(u_{r})+G(u_{r})\right] \right] \right| \\&\le \max _{\tau \in I}\left| N^{-1}\left[ \Big (\frac{v}{s}\Big )^{\mu }N^{+}[L(u_{p-1})-L(u_{q-1})+G(u_{p-1})-G(u_{q-1})]\right] \right| \\&\le \theta _1\max _{\tau \in I}\Big |N^{-1}\Big (\frac{v}{s}\Big )^{\mu }\Big [N^{+}[L(u_{p-1})-L(u_{q-1})]\Big ]\Big |\\&+\theta _2\max _{\tau \in I}\Big |N^{-1}\Big (\frac{v}{s}\Big )^{\mu }\Big [N^{+}[G(u_{p-1})-G(u_{q-1})]\Big ]\Big |\\&=(\theta _1+\theta _2)\frac{\tau ^{\mu }}{\Gamma (\mu +1)}\left\Vert u_{p-1}-u_{q-1}\right\Vert . \end{aligned} \end{aligned}$$

Consider $$p=q+1,$$ then

$$\begin{aligned} \begin{aligned} \left\Vert u_{q+1}-u_q\right\Vert&\le \theta \left\Vert u_q-u_{q-1}\right\Vert \\&\le \theta ^2\left\Vert u_{q-1}-u_{q-2}\right\Vert \\&\le \dots \le \theta ^q\left\Vert u_1-u_0\right\Vert , \end{aligned} \end{aligned}$$

where $\theta =(\theta _1+\theta _2)(1-\mu +\mu \tau ).$ Similarly we have

$$\begin{aligned} \begin{aligned} \left\Vert u_p-u_q\right\Vert&\le \left\Vert u_{q+1}-u_{q}\right\Vert +\left\Vert u_{q+2}-u_{q+1}\right\Vert +\dots +\left\Vert u_p-u_{p-1}\right\Vert \\&\le (\theta ^q+\theta ^{q+1}+\dots +\theta ^{p-1})\left\Vert u_1-u_0\right\Vert \\&\le \theta ^q\Big (\frac{1-\theta ^{p-q}}{1-\theta }\Big )\left\Vert u_1\right\Vert . \end{aligned} \end{aligned}$$

As $0<\theta <1,$ we obtain $1-\theta ^{p-q}<1.$ Then,

$$\begin{aligned} \left\Vert u_p-u_q\right\Vert \le \frac{\theta ^{q}}{1-\theta }\max _{\tau \in I}\left\Vert u_1\right\Vert . \end{aligned}$$

Since $\left\Vert u_1\right\Vert <\infty$. Thus, as $q\rightarrow \infty$, then $\left\Vert u_p-u_q\right\Vert \rightarrow 0$. As a results $u_p$ is a Cauchy sequence in E, the series $u_p$ is a convergent. $\square$

By following the above Theorem 2 procedure in a similar way, we can also prove that $NTDM_{CF}$ and $NTDM_{ABC}$ solutions are convergent.

## Numerical example of TFBBMBE

In this section, we will examine the approximate solutions to the TFBBMBE (1) along with the initial condition (2) as follows:

**NTDM**$_{C}$: By applying the natural transform decomposition technique on Eq. (1) with the Caputo fractional derivative, we obtain a series of consecutive solutions to the recurrent connection described in Eq. (16) as

$$\begin{aligned} {}^{C}u_{0}(\zeta ,\tau )&={{\,\textrm{sech}\,}}^2 \Big (\frac{\zeta }{4}\Big ),\\ {}^{C}u_{1}(\zeta ,\tau&)=\frac{\tau ^{{\mu }} \,\sinh \left( \frac{\zeta }{4}\right) \,{\left( {\cosh \left( \frac{\zeta }{4}\right) }^2 +1\right) }}{2\,{\Gamma }\left( {\mu }+1\right) \,{\cosh \left( \frac{\zeta }{4}\right) }^5 },\\ {}^{C}u_{2}(\zeta ,\tau )&=\frac{\tau ^{2\,{\mu }-1}}{128\,{\mu }\,{{\Gamma }\left( 2\,{\mu }\right) }^2 \,{\cosh ^8 \left( \frac{\zeta }{4}\right) } } \,\Big (10\,\tau \,{\Gamma }\left( 2\,{\mu }\right) \,\cosh \left( \frac{\zeta }{2}\right) -27\,\sinh \left( \frac{\zeta }{2}\right) \,\Gamma (2\mu +1)\\&\quad +4\,\cosh \left( \frac{\zeta }{2}\right) \,\sinh \left( \frac{\zeta }{2}\right) \,\Gamma (2\mu +1) +16\,\tau \,{\Gamma }\left( 2\,{\mu }\right) \,\cosh ^2\Big (\frac{\zeta }{2}\Big ) +2\,\tau \,{\Gamma }\left( 2\,{\mu }\right) \,{\cosh ^3 \left( \frac{\zeta }{2}\right) }\\&\quad +\cosh ^2\Big (\frac{\zeta }{2}\Big ) \,\sinh \left( \frac{\zeta }{2}\right) \,\Gamma (2\mu +1)-60\,\tau \,{\Gamma }\left( 2\,{\mu }\right) \Big ),\\&\quad \vdots \end{aligned}$$

we obtain the NTDM$_C$ series solution as follows:

$$\begin{aligned} {}^{C}u(\zeta ,\tau )&={{\,\textrm{sech}\,}}^2 \Big (\frac{\zeta }{4}\Big )+\frac{\tau ^{{\mu }} \,\sinh \left( \frac{\zeta }{4}\right) \,{\left( {\cosh \left( \frac{\zeta }{4}\right) }^2 +1\right) }}{2\,{\Gamma }\left( {\mu }+1\right) \,{\cosh \left( \frac{\zeta }{4}\right) }^5 }+\frac{\tau ^{2\,{\mu }-1}}{128\,{\mu }\,{{\Gamma }\left( 2\,{\mu }\right) }^2 \,{\cosh ^8 \left( \frac{\zeta }{4}\right) } } \,\\&\quad \times \Bigg (10\,\tau \,{\Gamma }\left( 2\,{\mu }\right) \,\cosh \left( \frac{\zeta }{2}\right) -27\,\sinh \left( \frac{\zeta }{2}\right) \,\Gamma (2\mu +1)+4\,\cosh \left( \frac{\zeta }{2}\right) \,\sinh \left( \frac{\zeta }{2}\right) \,\Gamma (2\mu +1) \\&\quad +16\,\tau \,{\Gamma }\left( 2\,{\mu }\right) \,\cosh ^2\Big (\frac{\zeta }{2}\Big ) +2\,\tau \,{\Gamma }\left( 2\,{\mu }\right) \,{\cosh ^3 \left( \frac{\zeta }{2}\right) } -60\,\tau \,{\Gamma }\left( 2\,{\mu }\right) +\cosh ^2\Big (\frac{\zeta }{2}\Big ) \,\sinh \left( \frac{\zeta }{2}\right) \,\Gamma (2\mu +1)\Bigg )+\cdots . \end{aligned}$$

**NTDM**$_{CF}$: By applying the natural transform decomposition technique on equation (1) with the CF derivative, we obtain a series of consecutive solutions to the recurrent connection described in equation (21) as

$$\begin{aligned} {}^{CF}u_{0}(\zeta ,\tau )&={{\,\textrm{sech}\,}}^2 \Big (\frac{\zeta }{4}\Big ),\\ {}^{CF}u_{1}(\zeta ,\tau )&=\frac{\sinh \left( \frac{\zeta }{4}\right) {\left( {\cosh ^2\left( \frac{\zeta }{4}\right) } +1\right) }{\left( 1-\mu +\mu \tau \right) }}{2{\cosh ^5\left( \frac{\zeta }{4}\right) } }, \end{aligned}$$

$$\begin{aligned} {}^{CF}u_{2}(\zeta ,\tau )&=\frac{1}{256~ \cosh ^{8}\Big (\frac{\zeta }{4}\Big )}\Bigg [416\mu (1-\tau +\mu \tau )-208(1+\mu ^2+2\mu ^2\tau ^2)\\&\quad +46 \cosh \Bigg (\frac{\zeta }{2}\Bigg )\Bigg (\mu ^2-1+2\mu \tau +\frac{\mu ^2\tau ^2}{2}\Bigg ) +107\mu \sinh \Bigg (\frac{\zeta }{2}\Bigg )(\mu -1-\mu \tau )\\&\quad +32\cosh (\zeta ) \Bigg ((1-\mu )^2+2\mu \tau (1-\mu )+\frac{\mu ^2\tau ^2}{2}\Bigg )+8\mu \sinh (\zeta )\Big (1-\mu +\mu \tau \Big )\\&\quad +\cosh \Bigg (\frac{3\zeta }{2}\Bigg )\Big (2(1+\mu ^2)+4\mu \tau (1-\mu )+\mu ^2\tau ^2\Big )+\sinh \Big (\frac{3\zeta }{2}\Big )\Big (1-\mu +\mu \tau \Big )\Bigg ],\\&\quad \vdots \end{aligned}$$

we obtain the NTDM$_{CF}$ series solution as follows:

$$\begin{aligned} {}^{CF}u(\zeta ,\tau )&={{\,\textrm{sech}\,}}^2 \Big (\frac{\zeta }{4}\Big )+\frac{\sinh \left( \frac{\zeta }{4}\right) \,{\left( {\cosh ^2\left( \frac{\zeta }{4}\right) } +1\right) }\,{\left( 1-\mu +\mu \,\tau \right) }}{2\,{\cosh ^5\left( \frac{\zeta }{4}\right) } }+\frac{1}{256~ \cosh ^{8}\Big (\frac{\zeta }{4}\Big )}\\&\quad \times \Big [416\,\mu \, (1-\tau +\mu \tau ) -208(1+\mu ^2+2\mu ^2\tau ^2)+107\mu \sinh \Big (\frac{\zeta }{2}\Big )(\mu -1-\mu \tau )\\&\quad +8\mu \sinh (\zeta )\Big (1-\mu +\mu \tau \Big ) +\cosh \Big (\frac{3\zeta }{2}\Big )\Big (2(1+\mu ^2)+4\,\mu \,\tau (1-\mu )+\mu ^2\tau ^2\Big )\\&\quad +46 \cosh \Big (\frac{\zeta }{2}\Big )\Big (\mu ^2-1+2\mu \tau +\frac{\mu ^2\tau ^2}{2}\Big )+\sinh \Big (\frac{3\zeta }{2}\Big )\Big (1-\mu +\mu \tau \Big ) \\&\quad +32\cosh (\zeta )\Big ((1-\mu )^2+2\mu \tau (1-\mu )+\frac{\mu ^2\tau ^2}{2}\Big )\Big ]+\cdots . \end{aligned}$$

**NTDM**$_{ABC}$: By applying the natural transform decomposition technique on Eq. (1) with the ABC derivative, we obtain a series of consecutive solutions to the recurrent connection described in Eq. (26) as

$$\begin{aligned} {}^{ABC}u_{0}(\zeta ,\tau )&={{\,\textrm{sech}\,}}^2 \Big (\frac{\zeta }{4}\Big ),\\ {}^{ABC}u_{1}(\zeta ,\tau )&=\frac{\sinh \left( \frac{\zeta }{4}\right) \,{\left( {\cosh ^2\left( \frac{\zeta }{4}\right) } +1\right) }}{2\,{\cosh ^5\left( \frac{\zeta }{4}\right) } }\Big [1-\mu +\mu \frac{\tau ^{\mu }}{\Gamma (1+\mu )}\Big ],\\ {}^{ABC}u_{2}(\zeta ,\tau )&=\frac{1}{512\,\tau \,(\Gamma (2\mu ))^2\,\Gamma (1+\mu )\,\cosh ^8\Big (\frac{\zeta }{4}\Big )}\Big [\mu \,(1-\mu )\,\tau ^{1+\mu }\,(\Gamma (2\mu ))^2\,\Big (184\,\cosh \,\Big (\frac{\zeta }{2}\Big )\\&\quad +8\cosh \Big (\frac{3\zeta }{2}\Big ) +128\cosh (\zeta )-832\Big )+\mu ^2(1-\mu )\tau ^{\mu }\,(\Gamma (2\mu ))^2\Big (-214\sinh \Big (\frac{\zeta }{2}\Big )\\&\quad +2\sinh \Big (\frac{3\zeta }{2}\Big )+16\sinh (\zeta )\Big ) +\mu \Gamma (1+\mu )\Gamma (1+2\mu )\tau ^{2\mu }\Big (-107\sinh \Big (\frac{\zeta }{2}\Big )\\&\quad +\sinh \Big (\frac{3\zeta }{2}\Big )+8\sinh (\zeta )\Big )+(\Gamma (2\mu ))^2\Gamma (1+\mu )\tau \Big (-416+64\cosh (\zeta )\\&\quad +92\cosh \Big (\frac{\zeta }{2}\Big )+4\cosh \Big (\frac{3\zeta }{2}\Big )\Big )+\mu (\Gamma (2\mu ))^2\Gamma (1+\mu )\Big (832-184\cosh \Big (\frac{\zeta }{2}\Big )\\&\quad -8\cosh \Big (\frac{3\zeta }{2}\Big )-128\cosh (\zeta )\Big )+\mu \Gamma (2\mu )\Gamma (1+\mu )\tau ^{1+2\mu }\Big (-208+46\cosh \Big (\frac{\zeta }{2}\Big )\\&\quad +2\cosh \Big (3\frac{\zeta }{2}\Big ) +32\cosh (\zeta )\Big )+\mu ^2(\Gamma (2\mu ))^2\Gamma (1+\mu )\tau \Big (-416+64\cosh (\zeta )\\&\quad +92\cosh \Big (\frac{\zeta }{2}\Big )+4\cosh \Big (\frac{3\zeta }{2}\Big )\Big )\Big ],\\&\quad \vdots \end{aligned}$$

we obtain the NTDM$_{ABC}$ series solution as follows:

$$\begin{aligned} {}^{ABC}u(\zeta ,\tau )&={{\,\textrm{sech}\,}}^2 \Big (\frac{\zeta }{4}\Big )+\frac{\sinh \left( \frac{\zeta }{4}\right) \,{\left( {\cosh ^2\left( \frac{\zeta }{4}\right) } +1\right) }}{2\,{\cosh ^5\left( \frac{\zeta }{4}\right) } }\Big [1-\mu +\mu \frac{\tau ^{\mu }}{\Gamma (1+\mu )}\Big ]\\&\quad +\frac{1}{512\,\tau \,(\Gamma (2\mu ))^2\,\Gamma (1+\mu )\,\cosh ^8\Big (\frac{\zeta }{4}\Big )}\Big [\mu \,(1-\mu )\,\tau ^{1+\mu }\,(\Gamma (2\mu ))^2\,\Big (184\,\cosh \,\Big (\frac{\zeta }{2}\Big )\\&\quad +8\cosh \Big (\frac{3\zeta }{2}\Big ) +128\cosh (\zeta )-832\Big )+\mu ^2(1-\mu )\tau ^{\mu }\,(\Gamma (2\mu ))^2\Big (-214\sinh \Big (\frac{\zeta }{2}\Big )\\&\quad +2\sinh \Big (\frac{3\zeta }{2}\Big )+16\sinh (\zeta )\Big ) +\mu \Gamma (1+\mu )\Gamma (1+2\mu )\tau ^{2\mu }\Big (-107\sinh \Big (\frac{\zeta }{2}\Big )\\&\quad +\sinh \Big (\frac{3\zeta }{2}\Big )+8\sinh (\zeta )\Big )+(\Gamma (2\mu ))^2\Gamma (1+\mu )\tau \Big (-416+64\cosh (\zeta )\\&\quad +92\cosh \Big (\frac{\zeta }{2}\Big )+4\cosh \Big (\frac{3\zeta }{2}\Big )\Big )+\mu (\Gamma (2\mu ))^2\Gamma (1+\mu )\Big (832-184\cosh \Big (\frac{\zeta }{2}\Big )\\&\quad -8\cosh \Big (\frac{3\zeta }{2}\Big )-128\cosh (\zeta )\Big )+\mu \Gamma (2\mu )\Gamma (1+\mu )\tau ^{1+2\mu }\Big (-208+46\cosh \Big (\frac{\zeta }{2}\Big )\\&\quad +2\cosh \Big (3\frac{\zeta }{2}\Big ) +32\cosh (\zeta )\Big )+\mu ^2(\Gamma (2\mu ))^2\Gamma (1+\mu )\tau \Big (-416+64\cosh (\zeta )\\&\quad +92\cosh \Big (\frac{\zeta }{2}\Big )+4\cosh \Big (\frac{3\zeta }{2}\Big )\Big )\Big ]+\cdots . \end{aligned}$$

**Table 1:** Absolute error of TFBBMBE for variation in $\zeta$ and $\tau$ with $\mu =1$.

$\zeta$	$\tau$	NTDM$_{C}$	NTDM$_{CF}$	NTDM$_{ABC}$	FHATM^28^	HPTT^21^
20	0.01	1.02220E−09	1.02220E−09	1.02220E−09	3.03359E−07	5.2389E−08
15	0.01	1.59373E−08	1.59373E−08	1.59373E−08	3.71650E−06	6.6306E−07
10	0.01	1.31338E−06	1.31338E−06	1.31338E−06	4.52894E−05	1.1546E−05
20	0.001	9.69493E−11	9.69493E−11	9.69493E−11	3.01818E−08	5.6476E$-$ $-$09
15	0.001	1.59271E−09	1.59271E−09	1.59271E−09	3.69715E−07	7.1250E−08
10	0.001	1.25714E−07	1.25714E−07	1.25714E−07	4.50091E−06	1.2098E−06

**Table 2:** Approximate solutions of TFBBMBE for variation in $\mu$, $\zeta$ and $\tau$.

$$\mu =0.8$$				
$\zeta$	$\tau$	NTDM$_{C}$	NTDM$_{CF}$	NTDM$_{ABC}$
20	0.01	1.8566E−04	2.0621E−04	2.1279E−04
15	0.01	2.2595E−03	2.5100E−03	2.5901E−03
10	0.01	2.7195E−02	3.0257E−02	3.1230E−02
20	0.001	1.8237E−04	2.0529E−04	2.1461E−04
15	0.001	2.2195E−03	2.4988E−03	2.6123E−03
10	0.001	2.6709E−02	3.0120E−02	3.1495E−02
$$\mu =0.9$$				
20	0.01	1.8370E−04	1.9425E−04	1.9613E−04
15	0.01	2.2357E−03	2.3642E−03	2.3871E−03
10	0.01	2.6906E−02	2.8473E−02	2.8752E−02
20	0.001	1.8187E−04	1.9327E−04	1.9520E−04
15	0.001	2.2134E−03	2.3523E−03	2.3758E−03
10	0.001	2.6634E−02	2.8327E−02	2.8612E−02

**Table 3:** Approximate solutions of TFBBMBE for various values of $\zeta$ and $\tau$ for $\mu =1$.

$\zeta$	$\tau$	NTDM$_{C}$	NTDM$_{CF}$	NTDM$_{ABC}$	Exact
20	0.01	1.82797E−04	1.82797E−04	1.82797E−04	1.82798E−04
15	0.01	2.22467E−03	2.22467E−03	2.22467E−03	2.22466E−03
10	0.01	2.67690E−02	2.67690E−02	2.67690E−02	2.67677E−02
20	0.001	1.81704E−04	1.81704E−04	1.81704E−04	1.81704E−04
15	0.001	2.21137E−03	2.21137E−03	2.21137E−03	2.21136E−03
10	0.001	2.66098E−02	2.66098E−02	2.66098E−02	2.66097E−02

**Figure 1:**
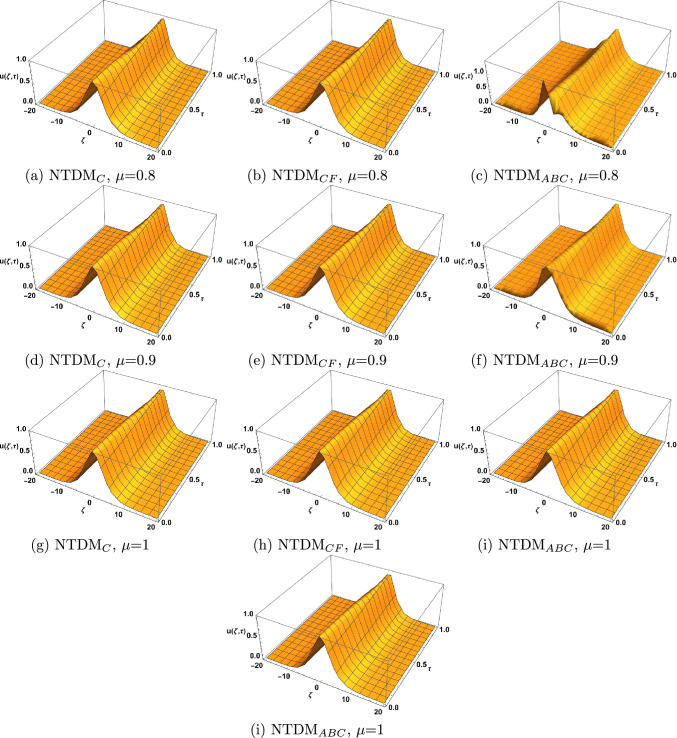
Surface plot for TFBBMBE with various values of $\mu$.

**Figure 2:**
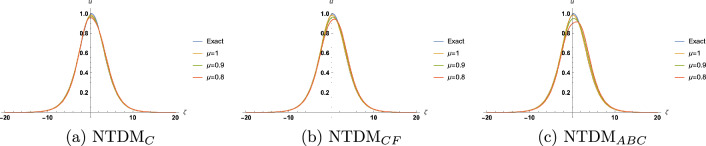
Approximate solution for TFBBMBE with various values of $\mu$ and $\tau =0.25$.

**Figure 3:**
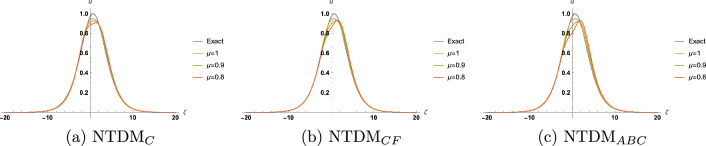
Approximate solution for TFBBMBE with various values of $\mu$ and $\tau =0.50$.

**Figure 4:**
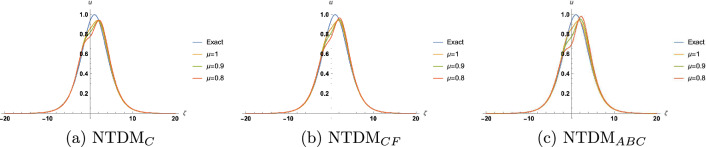
Approximate solution for TFBBMBE with various values of $\mu$ and $\tau =0.75$.

## Results and discussion

In this section, we present the solitary wave solutions of the time fractional Benjamin-Bona-Mahony-Burger equation that arise in the field of ocean engineering. This section presents TFBBMBE’s approximate solutions using NTDM, taking into account the fractional derivative in the Caputo, Caputo-Fabrizio, and ABC. The physical interpretation of the governing model is presented graphically with the help of 2D and 3D simulations. Hyperbolic functions were used to represent solitary wave solutions. We compared the proposed method solutions with the exact solutions through figures and tables. Table 1 displays a comparison of the absolute error of the proposed method with the existing methods in the literature, namely FHATM and HPTT, for variation in $\zeta$ and $\tau$ with $\mu =1$. Table 2 presents the approximate results of TFBBMBE for various values of $\mu$, $\zeta$, and $\tau$. A comparison of the approximate solutions of the proposed method for $\mu = 1$ with the exact solutions for various values of $\zeta$ and $\tau$ is given in Table 3. Tables 1 and3 demonstrate a strong agreement between the presented technique solutions and the exact solution, as well as the methods found in the literature, namely FHATM and HPTT. It can also be observed from the tables that, when the fractional order approaches 1, all three derivative solutions converge to the exact solution. Figure 1 demonstrates the 3D simulations of the proposed method solutions for various fractional orders for $-20\le \zeta \le 20$, and $0\le \tau \le 1$. Figures 2, 3 and 4 show the 2D graphical simulations of the approximate solutions of the TFBBMBE for various values of fractional orders at $\tau =0.25,0.50,0.75$, and $-20\le \zeta \le 20$. From Figs. 2, 3 and 4, it can be clearly seen that the accuracy decreases around $\zeta =0$. For large values of $|\zeta |$ in comparison with $\tau$, $\textrm{sech}^2\Big (\frac{\zeta }{4}-\frac{\tau }{3}\Big )$ tends to $\textrm{sech}^2\Big (\frac{\zeta }{4}\Big )$. Consequently, the $\tau$-induced error disappears. Conversely, when $\zeta$ approaches 0, the errors become evident. It is observed from the figures that the proposed method solutions with respect to C, CF, and ABC show similar behavior. The outcome of this study demonstrates the accuracy of the employed method. Therefore, the physical representation of our results has the potential to be a valuable tool for further investigation into nonlinear wave phenomena in scientific applications.

## Conclusion

This article presents the solitary wave solutions of the time fractional Benjamin-Bona-Mahony-Burger equation. The outcomes achieved through the utilization of the NTDM. Caputo, CF, and ABC fractional derivatives are used to characterize the fractional derivative.To validate the method, the outcomes of this study were presented in the form of tables and figures for various values of space and time variables by varying the fractional order. Various 2D and 3D graphical representations were utilized to analyze the impact of C, CF, and ABC derivatives on the dynamic behavior of the obtained solution. The methodology validates the accuracy and efficiency of the implemented method by comparing the obtained results with those of other methods in the existing literature, such as FHATM and HPTT. The results of the suggested method are consistent with those of these methods. This study may be helpful in investigating cracked rock phenomena, acoustic-gravity waves, acoustic waves, thermodynamics, diffusion theory, and wave propagation phenomena. The NTDM does not have any approximate functions or assumptions that could generate round-off errors. The NTDM is characterized by its straightforward principles and proven efficacy in solving nonlinear fractional differential equations. The study’s findings indicate that the strategy produces an approximation that nearly aligns to the exact solution, and that the approach is entirely in accordance with the exact solution. The major restriction that mimics the spread of flow rate is the value $\mu$, which is crucial for modeling unidirectional planar waves. The computational results are usually great, useful, and strong, and they can be expanded to provide more sophisticated, successful outcomes for several complex models from various applied scientific domains and engineering. The proposed approach can be utilized to address diverse models encountered in epidemiology, encompassing those pertaining to SIR model, Ebola, Zika virus, and Monkey Pox, as well as in the domains of science and engineering.

## Data Availability

The datasets used and analyzed during the current study available from the corresponding author on reasonable request.
